# Changes in thyroid fine needle aspiration practice during the COVID‐19 pandemic

**DOI:** 10.1111/cyt.13020

**Published:** 2021-07-12

**Authors:** Duccio Rossi, Alessia Belotti, Clementina di Tonno, Valeria Midolo, Fausto Antonio Maffini, Luca Nicosia, Elvio De Fiori, Giovanni Mauri

**Affiliations:** ^1^ Postgraduate School of Radiodiagnostics Università Degli Studi di Milano Milan Italy; ^2^ Postgraduate School of Pathology Università Degli Studi di Milano Milan Italy; ^3^ Division of Cytopathology IEO European Institute of Oncology IRCCS Milan Italy; ^4^ Division of Pathology IEO European Institute of Oncology IRCCS Milan Italy; ^5^ Department of Breast Radiology IEO European Institute of Oncology IRCCS Milan Italy; ^6^ Department of Radiology IEO European Institute of Oncology IRCCS Milan Italy; ^7^ Department of Oncology and Hematology‐Oncology University of Milan Milan Italy; ^8^ Division of Interventional Radiology IEO European Institute of Oncology IRCCS Milan Italy

**Keywords:** COVID‐19, cytology, fine needle aspiration, surgery, thyroid

## Abstract

**Purpose:**

To investigate the diagnostic accuracy of a different sample preparation protocol for fine needle aspiration cytology (FNAC) of thyroid nodules established during the COVID‐19 pandemic.

**Methods:**

From April 2020, conventional smears during FNAC were ceased according to World Health Organization recommendations due to the increased infection risk for operators, and a new protocol using only liquid‐based cytology (LBC) was adopted. FNACs performed between April and July 2020 (COVID‐19 group) were retrospectively compared with those from December 2019 through March 2020 (Pre‐COVID‐19 group). The distribution of diagnoses based on SIAPEC‐IAP categories and the concordance between cytological and histological results were compared using the chi‐squared test.

**Results:**

Categories based on FNAC for 90 and 82 thyroid nodules in the Pre‐COVID‐19 and COVID‐19 groups showed no significant difference in distribution (*P* = .081), with the following respective cases (and percentages): TIR1, 7 (8%) and 8 (10%); TIR1C, 0 (0%) and 6 (7%); TIR2, 59 (66%) and

55 (67%); TIR3A, 8 (9%) and 5 (6%); TIR3B, 1 (1%) and 2 (3%); TIR4, 5 (6%) and 1 (1%); and TIR5, 10 (12%) and 5 (7%). Among patients with potentially malignant lesions, surgery was performed for 12/16 (75%) nodules in the Pre‐COVID‐19 and 7/8 (88%) nodules in the COVID‐19 groups, with no significant differences between cytological and histological diagnoses (*P* = .931).

**Conclusion:**

The new LBC‐only protocol provided similar diagnostic accuracy in comparison with conventional smears, and can be effectively applied during a viral pandemic improving operator safety.

## Graphical abstract

The aim of this work was to investigate the diagnostic accuracy of a new sample preparation protocol for FNAC of thyroid nodules established during the COVID‐19 pandemic and to compare it with the diagnostic accuracy of the previously established standard protocol.

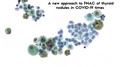

## 1 INTRODUCTION

Thyroid nodules are very common in the general population (being detected at rates of up to 65%), especially in women,^1^ and thyroid cancer is the most common neoplasm of the endocrine system, accounting for about 90% of cases.

Most thyroid lesions are benign and the malignant ones account for less than 10%,^2^ being usually well‐differentiated neoplasms with a slow growth and excellent outcomes. Papillary carcinoma is the most common form of well‐differentiated thyroid cancer.^3^, ^4^

The initial evaluation of a patient with thyroid nodules includes a full laboratory workup and ultrasound (US) evaluation with or without fine needle aspiration cytology (FNAC).

FNAC is a cost‐effective and useful method for assessing the nature of thyroid nodular lesions^5^ characterised by high sensitivity and many advantages such as ready‐to‐use equipment for a high‐quality product, a low rate of complications, and good patient tolerability.^6^

In most centres, the standard protocol for FNAC slide production consists of preparing thin smears that are rapidly air‐dried and/or fixed in 95% ethanol solution.^7^ Liquid‐based methods for cytology (LBC) have been reported to provide good diagnostic accuracy and are becoming routinely used, either alone or in combination with standard cytological preparation, especially to standardise the preanalytical procedures that are needed to perform immunocytochemistry (ICC) or molecular tests.^8^, ^9^, ^10^, ^11^ Even if there is still some debate regarding the routine application of LBC in thyroid FNAC, it should be noted that this method carries the not negligible advantage of also reducing the risk of aerosol diffusion of potentially infected material, which could be of particular value during a pandemic.

By April 2020, the coronavirus disease 2019 (COVID‐19)^12^ which was firstly reported in Wuhan, China,^13^ in December 2019, had dramatically spread in northern Italy. By then, stricter measures to address its specific biological risk in laboratories were being recommended.^14^ Due to the potential presence of the virus in histological and cytological specimens, the World Health Organization (WHO) advised that specimen processing should occur in accordance to biosafety level 2 guidelines.^15^, ^16^ Only tissue samples completely fixed in formalin (after 24 hours at room temperature) or in ethanol 95° were to be considered not a microbiological risk.^14^, ^17^, ^18^ COVID‐19 virus was identified mainly in samples of lung and oral origin, rarely in other biomaterials such as blood^19^ and faecal material.^20^ A recent work published at our institution demonstrated the presence of the virus even in the normal salivary gland samples using a real‐time PCR‐based assay. This result provided important information regarding possible infection sites or virus reservoirs and highlighted the need for proper fixation and handling prior to sample processing.^21^

Moreover, transmission of coronaviruses from contaminated inert surfaces was postulated, including self‐inoculation of mucous membranes of the nose, eyes, or mouth.^22^ According to a recent review article, the ﻿Severe Acute Respiratory Syndrome Coronavirus‐2 virus (SARS‐CoV‐2) can last on different surfaces between hours and a few days, though a rapid virus inactivation is possible using commonly available chemicals and biocides on dry surfaces.^23^ However, at our institution, from April 2020 a change in the FNAC protocol was decided, avoiding both air‐dried and alcohol‐fixed slides. To avert potentially infectious aerosol formation, all biological material must be fixed in an alcohol‐based fixative solution. The adoption of LBC with a ThinPrep^®^ automated system was decided according to our security criteria.

The aim of this work is to report the diagnostic accuracy of this new sample preparation protocol for FNAC of thyroid nodules established during the COVID‐19 pandemic and to compare it with the diagnostic accuracy of the previously established standard protocol.

## 2 METHODS

Approval for this retrospective study was obtained from our institution’s Ethics Committee and patients' informed consent was waived. All thyroid US‐guided FNACs for suspicious lesions performed between December 2019 and July 2020 at our institution were retrospectively analysed.

From the 4th of April, air‐dried or alcohol‐fixed slides were no longer used at our institution being high in risk of infectious aerosol formation while performing all the cyto‐preparatory steps. Thus, we consider the 4th of April as the watershed day between the old and new protocols, dividing patients into two groups according to a specific time‐based distinction labelled “Pre‐COVID‐19” (3 December 2019–3 April 2020) and “COVID‐19” (4 April 2020–31 July 2020).

Cytological characterisation of lesions was reported, according to the joint classification of the Italian Society for Anatomic Pathology and Cytology with the Italian Division of the International Academy of Pathology (SIAPEC‐IAP), as TIR1 (Inadequate), TIR1C (Inadequate‐cystic), TIR2 (Benign), TIR3A (Indeterminate lesion with low risk of malignancy), TIR3B (Indeterminate lesion with high risk of malignancy), TIR4 (Suspicious for malignancy), and TIR5 (Malignant).^24^

Surgery was performed when high‐risk or malignant lesions (TIR3B, TIR4, TIR5) were detected. The final diagnosis on histopathological reports was recorded when surgery was carried out at our institution.

All FNAC procedures were US‐guided. Following identification of the lesion by ultrasonography and skin disinfection with alcohol, a 25‐gauge needle was inserted into the desired nodule with the no‐aspiration technique, according to which the needle was moved in different directions for a few seconds allowing material to enter it by capillary action. In the Pre‐COVID‐19 period, the needle content was expelled onto previously labelled slides and the smear was simply prepared by touching the second slide to the surface and separating them again. One set of slides was placed in the holder containing 95% alcohol solution while another set was left to air‐dry. Another tissue sample was then performed on the desired nodule using a 22‐gauge needle connected to a 20‐mL syringe with the aspiration technique, according to which the negative pressure created by the pulling back of the syringe plunger was useful to collect cells into the cutting edge of the needle. The material was immediately rinsed into a ready‐to‐use ThinPrep CytoLyt^®^ (Hologic Corporation) alcohol‐based fixative solution following the LBC method. The slide containers, labelled with the patient data, were sent to the cytology laboratory, along with a complete Cytopathology Requisition Form, including pertinent patient history.

During the COVID‐19 period, in addition to the use of personal protective equipment including FFP2 or FFP3 masks, protective glasses and gloves, air‐dried or alcohol‐fixed slides and all air‐dried handling materials were avoided and only the ThinPrep CytoLyt solution and LBC method were adopted. The ThinPrep CytoLyt solution was used to maximise the reduction in contamination risk without the need of further material handling after the FNA procedure. Therefore, the adoption of the CytoLyt solution and the LBC method allowed for both optimal morphological tissue preservation and reduction in the aerosol and droplet formation during conventional slide preparation.

According to this new protocol, the material was directly processed in the Cytopathology Laboratory, in a dedicated high‐level biosafe hood as specified below:

1. The cytological specimen was put in a PreservCyt^®^ solution vial and allowed to stand for at least 15 minutes (this step is fundamental to guaranteeing the complete SARS‐CoV‐2 virus inactivation, as instructed in the Technical Bulletin issued by the production house).
2. If the material in the vial appeared strongly turbid or bloody, it was washed with acetic acid and CytoLyt solutions before being prepared for the ThinPrep slides and run on a ThinPrep 5000 processor. If the specimen appeared clear, the ThinPrep slides were directly prepared and run on a ThinPrep 5000 processor.

### 2.1 Data analysis

The distribution of the diagnostic categories between the Pre‐COVID‐19 and COVID‐19 group was evaluated. When considering high‐risk or malignant lesions, we also evaluated the accuracy and reliability of the Pre‐COVID‐19 and COVID‐19 protocols using subsequent diagnosis on surgical specimens as reference when available.

Continuous data are reported as median values and ranges. Categorical data are reported as counts and percentages. Pearson's chi‐squared test was used to assess the distribution of the diagnostic categories between the two groups and to evaluate the agreement of cytological and histological diagnoses of potentially malignant lesions between the two methods. *P* values less than .05 were considered statistically significant. All data were collected and analysed on a Microsoft Excel spreadsheet.

## 3 RESULTS

During the Pre‐COVID‐19 period 90 FNAC thyroid procedures were performed on 88 patients, compared with 82 procedures on 81 patients conducted during the COVID‐19 period.

Patients' average age was 57 years for the Pre‐COVID‐19 group and 53 for the COVID‐19 group (range 22‐81 years and 24‐78 years, respectively). SIAPEC‐IAP classification for samples in the Pre‐COVID‐19 group and COVID‐19 group, respectively, were as follows: TIR1 in 7/90 (8%) and 8/82 (10%), TIR1C in 0/90 (0%) and 6/82 (7%), TIR2 in 59/90 (66%) and 55/82 (67%), TIR3A 8/90 (9%) and 5/82 (6%), TIR3B 1/90 (1%) and 2/82 (3%), TIR4 5/90 (6%) and 1/82 (1%), TIR5 10/90 (12%) and 5/82 (7%). No statistically significant difference was found in the distribution of cases among diagnostic categories (*P* = .081; Table 1). Among the high‐risk patients (TIR3, TIR4, TIR5), 12/16 (75%) in the Pre‐COVID group and 7/8 (88%) in the COVID group underwent surgery at our institution, and no statistically significant difference was found between the two groups in terms of agreement between cytological and histological diagnoses of potentially malignant lesions (*P* = .931) (Table 2).

**TABLE 1:** Distribution of diagnostic categories between the Pre‐COVID‐19 group (n = 90) and the COVID‐19 group (n = 82)

FNAC diagnoses	Pre‐COVID‐19 (n = 90)	COVID‐19 (n = 82)
TIR1	7 (8%)	8 (10%)
TIR1C	0 (0%)	6 (7%)
TIR2	59 (66%)	55 (67%)
TIR3A	8 (9%)	5 (6%)
TIR3B	1 (1%)	2 (2%)
TIR4	5 (6%)	1 (1%)
TIR5	10 (11%)	5 (6%)
*P* value <.05	.081^a^	

^a^ Chi‐squared test. Abbreviations: FNAC, fine needle aspiration cytology; TIR1, Inadequate; TIR1C, Inadequate‐cystic; TIR2, Benign; TIR3A, Indeterminate lesion with low risk of malignancy; TIR3B, Indeterminate lesion with high risk of malignancy; TIR4, Suspicious for malignancy; TIR5, Malignant.

**TABLE 2:** Comparison of cytological and histological diagnoses for potentially malignant lesions (TIR3B, TIR4, TIR5) in patients of the Pre‐COVID‐19 (n = 12/16; 75%) and COVID‐19 groups (n = 7/8; 88%) who underwent surgery at our institution

Cytology	Histology			
	Pre‐COVID‐19 (n = 12)		COVID‐19 (n = 7)	
	Benign	Carcinoma	Benign	Carcinoma
TIR3B	0	0	2 (100%)	0
TIR4	1 (100%)	2 (18%)	0	1 (20%)
TIR5	0	9 (82%)	0	4 (80%)
*P* value <.05	.931^a^			

^a^ Chi‐squared test. Abbreviations: TIR3B, Indeterminate lesion with high risk of malignancy; TIR4, Suspicious for malignancy; TIR5, Malignant.

## 4 DISCUSSION

US‐guided FNAC has an essential role in the diagnostic pathway of thyroid nodules especially for its availability, rapidity, cost‐effectiveness, and low level of associated procedural risks.

Air‐dried slides with Romanowsky stain (Diff‐Quik, May‐Grünwald‐Giemsa) usually represent a ﻿rapid and useful method to enhance pleomorphism and distinguish extracellular from intracytoplasmic material allowing good definition of the cell outline and cytoplasmic contents,^25^ whereas alcohol‐fixed slides with Papanicolaou (Pap) stain allow a clearer visualisation of the cellular morphology and nuclear features.

However, the extraordinary COVID‐19 emergency forced us to rethink the organisation and practices of FNAC considering that, according to recommendations of the WHO and other international organisations, the slide preparation could be dangerous because of the potentially infectious material.^15^, ^16^

The aim of our work was to show the preliminary results of FNAC performed by using exclusively an LBC method that differs from the traditional one based on the preparation of cytological smears (CSs).

The overall number of thyroid FNAC procedures during the COVID‐19 period was slightly lower compared to that of the Pre‐COVID‐19 period (82 vs 90, respectively), clearly related to the restrictions imposed by the Italian Government.

Our results showed no significant difference between the two types of procedures. Occasionally, more patients with purely cystic nodules presented during the COVID‐19 period and this could explain the relatively higher proportion of TIR1C samples in this group. In this case, the final diagnosis of completely cystic nodule was made through the correlation of cytological and radiological features. However, even accounting for the higher number of patients with purely cystic nodules in the COVID‐19 group, no statistically significant difference in the distribution of diagnostic categories between the two groups was found (*P* = .081). Also, for lesions suggestive of malignancy (TIR3B, TIR4 and TIR5), there was no statistically significant difference in the agreement between cytological and histological results for both protocols (*P* = .931).

The LBC method differs from the traditional one for a distinctive fixative method without a staining process that potentially removes diagnostic features as necrotic debris or colloid. The use of LBC‐only versus CSs for thyroid FNA specimens is a long‐standing dispute, especially because cytopathologists have always used CS slides and therefore are more used to the cytomorphological features of thyroid lesions.^8^, ^9^, ^10^, ^11^ Recent studies demonstrated that an LBC‐only method performs better than CS in terms of sample adequacy and it is almost the same in terms of sensitivity and specificity.^26^ The main advantages of LBC consist of its simple handling, excellent storage of the material,^9^, ^27^ and faster microscopic examination of the samples as the cells are concentrated in a limited area and no air‐drying artifacts are present.^28^ However, whereas the FNAC diagnosis of thyroid nodules is based on the observation of how the cells are clustered and on background materials such as colloid, the LBC method causes disruption of cell clusters, colloid fragmentation, and removal of background material. This is more evident for papillary lesions in which intranuclear pseudoinclusions, frequently indicative of malignancy, are less clear in LBC than in CSs. On the other hand, other characteristics of benign follicular lesions such as macrofollicular architecture are similar in both methods.^26^ However, as recently showed by Straccia et al^29^ in a paper where an LBC protocol similar to ours was adopted for the evaluation of all cytological samples, the morphological details and quality of the cellular component can be effectively preserved to achieve good diagnostic efficacy.

In the future, another approach, based on cytology combined with molecular testing on an LBC method, could ﻿improve the diagnosis of indeterminate FNAs, still avoiding the use of CSs.^30^, ^31^, ^32^

Our study has some limitations, mainly its retrospective nature and the fact that from a pathologist's perspective, common Papanicolaou and Romanowsky stains on CSs are still used in a complementary fashion and currently represent the mainstay in the approach to the FNAC diagnosis.^8^ However, our approach, developed in a case of extreme emergency, suggests the reliability of an LBC method in terms of diagnosis and safety for healthcare workers.

## 5 CONCLUSIONS

As the COVID‐19 pandemic is still ongoing and vaccines, social‐distancing, and contact prevention hold a crucial role in controlling the disease, the routine application of LBC to thyroid FNAC increases the safety of procedures without compromising diagnostic accuracy. Thus, our work supports a larger application of LBC, which could be particularly useful in case of a viral pandemic, being a reliable procedure while reducing the risk of viral spread.

## CONFLICT OF INTEREST

The authors declare there are no conflicts of interest.

## AUTHOR CONTRIBUTIONS

DR, AB, C.d.T., VM, FAM, LN, E.d.F., G. M. contributed to the design and implementation of the research and to the writing of the manuscript. All authors have read and agreed to the published version of the manuscript.

## ETHICAL APPROVAL

All procedures performed in studies involving human participants were in accordance with the ethical standards of the institutional and/or national research committee and with the 1964 Helsinki declaration and its later amendments or comparable ethical standards.

## INFORMED CONSENT

Informed consent was obtained from all individual participants included in the study.

## Data Availability

The data that support the findings of this study are available on request from the corresponding author.
